# Measuring Changes in Perceptions of Access to Pet Support Care in Underserved Communities

**DOI:** 10.3389/fvets.2021.745345

**Published:** 2021-12-10

**Authors:** Sloane M. Hawes, Tess M. Hupe, Jordan Winczewski, Kaitlyn Elting, Amanda Arrington, Sandra Newbury, Kevin N. Morris

**Affiliations:** ^1^Institute for Human-Animal Connection, Graduate School of Social Work, University of Denver, Denver, CO, United States; ^2^Pets for Life, The Humane Society of the United States, Gaithersburg, MD, United States; ^3^Shelter Medicine, School of Veterinary Medicine, University of Wisconsin - Madison, Madison, WI, United States

**Keywords:** companion animals, access to care, animal welfare, social determinants of health, generalized estimating equations

## Abstract

Understanding social, economic, and structural barriers to accessing pet care services is important for improving the health and welfare of companion animals in underserved communities in the U.S. From May 2018-December 2019, six questions from the validated One Health Community Assessment were used to measure perceptions of access to pet care in two urban and two rural zip codes. One urban and one rural community received services from a pet support outreach program (Pets for Life), while the other served as a comparison community. After propensity score matching was performed to eliminate demographic bias in the sample (Urban = 512 participants, Rural = 234 participants), Generalized Estimating Equations were employed to compare the six measures of access to pet care between the intervention and comparison communities. The urban community with the Pets for Life intervention was associated with a higher overall measure of access to pet care compared to the urban site that did not have the Pets for Life intervention. When assessing each of the six measures of access to care, the urban community with the Pets for Life intervention was associated with higher access to affordable pet care options and higher access to pet care service providers who offer payment options than the community without the Pets for Life intervention. Further analyses with a subset of Pets for Life clients comparing pre-intervention and post-intervention survey responses revealed statistically significant positive trends in perceptions of two of the six measures of access to pet care. This study provides evidence that community-based animal welfare programming has the potential to increase perceptions of access to pet support services.

## Introduction

Access to veterinary care and other pet supportive services (e.g., grooming, behavior training, pet supplies) has been increasingly recognized within the animal welfare sector as a substantial barrier to the health and welfare of companion animals (henceforth referred to as “pets”). Early academic definitions of access to care in the human health sector consisted of five dimensions, including: availability (e.g., the quantity and types of services); accessibility (e.g., the geographic location); accommodation (e.g., the hours of operation, service models, and facility types); affordability (e.g., options for low-cost services and insurance coverage); and acceptability (e.g., high quality services that consider a client's unique preferences or needs) (1). However, developing programs that address all five of these dimensions are likely insufficient without also incorporating the important distinction between an individual “having access” to services, meaning an individual has the potential to access a particular service, and an individual “gaining access,” referring to an individual's actual utilization of the service (2). Within this broadened definition, an individual's ability to “gain access” depends on additional social and community factors included in the social determinants of health framework (2).

The Centers for Disease Control and Prevention, defines social determinants of health as “conditions in the places where people live, learn, work, and play that affect a wide range of health risks and outcomes” (3). These determinants include an individual's social and community context, economic stability, neighborhood and built environment, education access and quality, and healthcare access and quality. There have been increased efforts to improve human health trajectories by addressing the influence of social determinants of health. These efforts are operationalized as interventions to address a number of potential systemic barriers to accessing human healthcare, including: housing and built environment (e.g., Gautreaux Residential Mobility Program, Healthy Food Financing Initiative, Project U-Turn; Scattered-Site Public Housing Program, Moving to Opportunity for Fair Housing Demonstration Program); low socioeconomic status (e.g., Great Smoky Mountain Study, Supplemental Security Income Program, New Hope Random Assignment Experiment, Conditional Cash Transfer Programs, Special Supplemental Nutrition Program for Women, Infants and Children, Earned Income Tax Credit); education (e.g., Perry Preschool Project, Carolina Abecedarian Project, Nurse Family Partnership, Harlem Children's Zone); and employment (e.g., Civil Rights Policies, Supported Employment). Research indicates that these programs have resulted in reduced health disparities, improved population health, decreased morbidity and mortality, and lower medical care costs in historically marginalized communities (e.g., Black, Indigenous, or People of Color, LGBTQ+ individuals, individuals living in poverty or experiencing homelessness, individuals with disabilities, and aging adults) (4–6).

Like human healthcare, social determinants of health, such as access to care, also impact the health and welfare of pets. Several factors that inform access to pet support services have been identified, including service provider-client relationships and communication, cultural or language barriers, client perceptions of the necessity of veterinary and other pet support services, transportation barriers, clinic hours of operation, a client's disability or medical condition, client education, and affordability of services (7–10). In a recent study, qualitative interviews with pet-owning residents in a community with low socioeconomic status identified affordability of pet care services, geographic proximity to pet care services, availability of pet care services in an individual's preferred language, and access to pet care information as the most important components of accessing pet supportive programming (11). Among these barriers, affordability is the most frequently discussed in current literature, with over 25 previous studies focusing on this challenge [e.g., (7–9, 11–19)]. A number of programs have been initiated to improve access to basic veterinary care and pet support services (8, 20–31); including service-learning programs that strive to prepare veterinary students to address barriers to accessing pet care (10). Unfortunately, many of these programs view barriers to accessing care as a personal issue, opt to address only one dimension of access (e.g., affordability, geographic accessibility), or determine program efficacy by evaluating just one measurement of success, such as number of services provided (12). Furthermore, research examining the efficacy of interventions addressing the social determinants of health that disproportionately impact pets and their owners in historically marginalized communities is still limited. However, it is likely that incorporating an understanding of both the individual and structural factors that inform human health outcomes in historically marginalized communities into the development of pet support service programs will improve animal welfare organizations' engagement with these traditionally underserved populations.

One of the most well-established and longest running programs to improve access to pet support services in historically marginalized communities is The Humane Society of the United States' Pets for Life (PFL) program. PFL addresses the issue of access to pet support services by offering no cost or heavily subsidized pet care services, providing transportation to and from appointments, employing bilingual staff members, building relationships with pet owners in the community, and partnering with local companion animal service organizations to provide services. Since 2011, PFL has served over 200,000 pets by providing over 600,000 veterinary services, supplies, and medications in 50 communities in the U.S. and Canada (32). The PFL model provides an opportunity to study the impacts of community-based animal welfare programming. In the present study, questions from the One Health Community Assessment (OHCA) instrument were used to evaluate community members' perceptions of their access to pet support services. It was hypothesized that community members in historically underserved communities that received the PFL intervention would have more positive perceptions of their access to pet support services than community members living in a similar community that was not receiving PFL services.

## Materials and Methods

### Data Collection

The data for this study were collected as part of an ongoing four-year study to assess the impacts of the PFL intervention in historically underserved communities. To assess the effectiveness of PFL in addressing access to pet support services, four communities (comprised of single zip codes) were selected for the study. Several factors impacted the study site selection criteria. First, due to the regional focus of the funder, only eight states (AK, ID, MT, MN, ND, OR, WA, WI) were considered in the selection process. Second, communities were evaluated based on meeting the specific criteria describing an underserved community. These criteria included the absence of local veterinarians and pet service providers (e.g., pet supply stores). Geographic Information System (GIS) mapping of veterinary clinics and other pet service providers listed in local business registries was used to determine the study communities' limited geographic proximity to pet care resources (ArcGIS—Environmental Systems Research Institute, Redlands, CA, USA). Third, demographic factors (e.g., median household income, poverty rate, unemployment rate) were evaluated amongst the list of communities to identify similarities. Within the eight states, this narrowed the search to 27 rural communities and 30 urban communities. An urban community was defined as an area within a large city that contains highly concentrated residential and commercial properties, and a rural community was defined as a region of undeveloped land with a low population size and density (33).

To allow for an initial assessment of the generalizability of findings across communities, the four study sites included two urban and two rural communities. The pair of urban study sites chosen were in Madison, WI (53713) and Seattle, WA (98108), and the pair of rural sites were Granger, WA (98932) and Wilder, ID (83676). When selected for the study in 2017, the urban and rural pairs were found to have similarities across the following demographic characteristics: population size, race/ethnicity composition, poverty rate, and median household income level [Table 1; (34)]. Each site has a greater number of households living below the federal poverty line and higher racial and ethnic diversity than the U.S. averages (34). To understand the total number of pets who could potentially benefit from PFL services, a detailed assessment of pet ownership was conducted during the first year of the study in each of the four study communities. The measured rate of pet ownership in each of the study sites was: Madison 58.6%, Seattle 48.1%, Granger 64.7%, and Wilder 64.9% (35). Using a wait list control design, one site in each of the pairs received the PFL intervention (Madison, WI and Granger, WA), while the other site served as a comparison community (Seattle, WA and Wilder, ID).

**Table 1:** 2017 Demographic data of the four study communities (34).

**Study site**	**Population (2017)**	**Ethnicity**							**Median household income**	**Percentage of individuals below the poverty level**
		**Native American**	**Asian**	**Black**	**Latino/a**	**White**	**Multi-ethnic**	**Other**		
Granger, WA (98932)	5,335	2.9%	0.3%	0.9%	76.7%	17.6%	1.6%	0%	$47,302	27.3%
Wilder, ID (83676)	4,511	0.3%	0.2%	0%	35.7%	62.5%	1.0%	0.2%	$45,645	15.4%
Seattle, WA (98108)	24,134	0.6%	37%	18.7%	10.1%	26.4%	5.8%	1.3%	$55,314	23.3%
Madison, WI (53713)	23,097	0.6%	7.6%	15.6%	25.6%	46%	4.5%	0.1%	$38,843	27.8%

The data for this study were collected in each of the study sites by grant-funded community-based research assistants (CBRAs) following a University of Denver IRB approved consent and data collection protocol (DU IRB protocol 1234950). The CBRAs were employed by the local animal welfare organizations (Dane County Humane Society for Madison, WI, Seattle Humane for Seattle, WA, Yakima Humane Society for Granger, WA, and Idaho Humane Society for Wilder, ID) and received intensive training on culturally appropriate research methods from the research team. Regular fidelity checks were conducted with each of the CBRAs to ensure data collection was implemented consistently across the four study communities. Fidelity checks were conducted by full time research staff members at the University of Denver, who have prior experience and certification in conducting survey-based research and were responsible for designing this study. These fidelity checks were conducted yearly with the CBRAs. During a fidelity check, the research staff member observed a CBRA provide an explanation of the study, execute the informed consent process, and conduct the survey. Some of the key areas assessed during a fidelity check included the research staff members' ability to build rapport with the community member, their accuracy in reading the questions, and their explanation and reporting of the Likert scale responses. The research staff member provided coaching and feedback to the CBRA for improvement in the future. The CBRAs live in or near their focus community and were hired based on their previous experience in community-based data collection, including their skills in building rapport with diverse community members. The CBRAs collected the data using systematic sampling grids to guide their door-to-door recruitment efforts. These systematic sampling grids included half of the households in the urban communities and all of the households in the rural communities. To maximize response rates, CBRAs made three contact attempts at every household, with each attempt occurring on different days of the week and times of day. When contact was established at a household, the CBRA explained the study goals and assessed if the resident met the inclusion criteria.

The inclusion criteria for individuals participating in the study included: living in a household within one of the four study community zip codes (53713, 98108, 98932, 83676) and if they currently owned pets or had owned pets within the previous 12 months. For those who were eligible and consented to participate, the CBRA began by collecting human and pet demographic data. This included information about the pet owner's household income, ethnicity, and housing type, and data on their pet(s) names, type and breed of their pet(s), and where they obtained their pet(s) from. The CBRA then administered the OHCA instrument. The OHCA is a validated instrument measuring community members' perception of numerous factors contributing to community-wide One Health (Cronbach's alpha = 0.9, 11), the interconnected health of people, other animals, and the environment (36). This instrument was developed using an exploratory sequential mixed methods approach and piloted with over 100 community members in a PFL community in Denver, CO. In the present study, six of the 115 questions from the OHCA were used to assess components of access to care, including an individual's perceptions of the affordability of their pet care services (e.g., veterinarians, grooming, behavior support, pet supply retailers), geographic proximity to pet care services, availability of services in the individual's preferred language, and availability of information regarding pet healthcare. The questions were structured on a 5-point Likert scale with 1 = “Strongly Disagree,” 2 = “Disagree,” 3 = “Neutral,” 4 = “Agree,” 5 = “Strongly Agree,” and a response option for “prefer not to answer.” CBRAs asked all questions verbally in the preferred language of the participant (Spanish or English). All responses were entered using electronic tablets into a HIPAA-compliant data management system hosted at the University of Denver (REDCap) (37).

Two time points of data collected from the individuals living in the study communities were analyzed in this study. The first time point was collected from May 2018 to April 2019 when the PFL interventions were initiated in Madison, WI and Granger, WA, and the second time point was collected from May 2019 to December 2019. Data from the intervention sites (Madison, WI and Granger, WA) and comparison sites (Seattle, WA and Wilder, ID) were used to explore how the presence of PFL in a community (but not necessarily direct participation as a PFL client) influences measures of access to pet care. Participating households were included in the analyses if they completed at least one time point of data collection during the study period.

### Propensity Score Matching

The PSmatching3 tool in Statistical Package for the Social Science (SPSS) version 25 was used to execute propensity score matching of the dataset to create balance in respect to potentially confounding demographic variables between the intervention and comparison communities. Propensity score matching is used to reduce bias in a study's assessment of how the intervention (presence of PFL in a community) impacts the measured outcome (perceptions of access to pet care) (38). The propensity score is a balancing score, which allows a nonrandomized study design to mimic some characteristics of a randomized control trial. The demographic variables included in the propensity score matching were gender, ethnicity, age, preferred language, household income, highest level of education completed, born in the U.S., and current housing status. Multiple propensity score models (matching order largest, smallest, and random) were tested with the 1-to-1 nearest neighbor approach (caliper 0.2), and the model with the best overall balance was selected to estimate the intervention effect. Demographics of the sample before and after propensity score matching can be found in Table 2.

**Table 2:** Demographics of the sample before and after propensity score matching.

	**Pre-PSM: urban sites** **(*N* = 598)**	**Pre-PSM: rural sites** **(*N* = 404)**	**Post-PSM: urban sites** **(*N* = 512)**	**Post-PSM: rural sites** **(*N* = 234)**
**PFL**				
Intervention group	299 (50%)	238 (58.9%)	256 (50%)	117 (50%)
Comparison group	299 (50%)	166 (41.1%)	256 (50%)	117 (50%)
**Preferred language**				
English	565 (94.5%)	299 (74%)	488 (95.3%)	189 (80.8%)
Spanish	22 (3.7%)	105 (26%)	19 (3.7%)	45 (19.2%)
Other	8 (1.3%)	0	5 (1%)	0
Prefer not to answer	3 (0.5%)	0	0	0
**Sex**				
Male	238 (39.8%)	124 (30.7%)	206 (40.2%)	66 (28.2%)
Female	351 (58.7%)	280 (69.3%)	301 (58.8%)	168 (71.8%)
Other	3 (0.5%)	0	2 (0.4%)	0
Prefer not to answer	6 (1%)	0	3 (0.6%)	0
**Age (years)**				
60 or older	113 (18.9%)	95 (23.5%)	95 (18.6%)	61 (21.6%)
30–60	358 (59.9%)	194 (48%)	309 (60.4%)	103 (44%)
18–30	124 (20.7%)	109 (27%)	107 (20.9%)	67 (28.6%)
Prefer not to answer	3 (0.5%)	6 (1.5%)	1 (0.2%)	3 (1.3%)
**Ethnicity**				
White	387 (64.7%)	140 (34.7%)	347 (67.8%)	89 (38%)
Latino/a	54 (9%)	234 (57.9%)	46 (9%)	129 (55.1%)
Black	75 (12.5%)	2 (0.5%)	67 (13.1%)	2 (0.9%)
Other (Asian, Native American, multi-ethnic)	78 (13%)	27 (6.7%)	51 (10%)	14 (6%)
Prefer not to answer	4 (0.8%)	1 (0.2%)	1 (0.2%)	0
**Household income ($)**				
0–15,000	88 (14.7%)	52 (12.9%)	80 (15.6%)	32 (13.7%)
15,000–30,000	74 (12.4%)	70 (17.3%)	69 (13.5%)	39 (16.7%)
30,000–45,000	61 (10.2%)	73 (18.1%)	50 (9.8%)	42 (17.9%)
45,000–60,000	66 (11%)	42 (10.4%)	57 (11.1%)	20 (8.5%)
60,000 or more	207 (34.6%)	64 (15.8%)	179 (35%)	44 (18.8%)
Prefer not to answer	102 (17.1%)	103 (25.5%)	77 (15.1%)	57 (24.4%)
**Education**				
Less than a high school degree	52 (8.7%)	97 (24%)	46 (9%)	50 (21.4%)
High school degree or equivalent	291 (48.7%)	241 (59.7%)	249 (48.6%)	144 (61.5%)
College degree	244 (40.8%)	58 (14.4%)	209 (40.8%)	35 (15%)
Prefer not to answer	11 (1.8%)	8 (1.9%)	8 (1.6%)	5 (2.1%)
**Housing status**				
Homeowner	146 (24.4%)	113 (28%)	123 (24%)	76 (32.5%)
Renter	75 (12.5%)	51 (12.6%)	66 (12.9%)	29 (12.4%)
Unstably housed	9 (1.5%)	19 (4.7%)	8 (1.6%)	13 (5.6%)
Other	8 (1.3%)	2 (0.5%)	7 (1.4%)	2 (0.9%)
Prefer not to answer	360 (60.2%)	219 (54.2%)	308 (60.1%)	114 (48.6%)
**Born in the U.S**.				
Yes	523 (87.5%)	284 (70.3%)	463 (90.4%)	178 (76.1%)
No	72 (12%)	115 (28.5%)	47 (9.2%)	55 (23.5%)
Prefer not to answer	3 (0.5%)	5 (1.2%)	2 (0.4%)	1 (0.4%)

### Exploratory Analyses

Missing data were common for participants in this dataset due to challenges associated with conducting door-to-door data collection. In the urban sites, 337 (65.8%) participants completed the survey for one time-point only (year one or year two). In the rural sites, 127 (54.3%) participants completed the survey for one time-point only (year one or year two). To assess if the missingness mechanism differed between the intervention and comparison communities, Little's Missing Completely at Random (MCAR) tests were performed using data on the availability to complete a follow-up survey after year one. This test assesses whether the missingness depends on the observed and unobserved variables within the dataset (39). Results of Little's MCAR tests provided reason to reject the null hypothesis that the data were missing completely at random in the urban sites (*p* < 0.001) and rural sites (*p* = 0.001), respectively. Additional analysis using Chi-Square tests comparing the availability for a follow-up survey after the first year of data collection demonstrated that there was no significant difference in missingness proportions between the two urban communities. This point of similarity in availability for follow-up supports the assumption that comparison of the response variables over time between these communities is not biased by differing availability for follow-up. However, the Chi-Square test reflected differences in availability to follow-up in the rural sites, with survey participants in Wilder, ID associated with greater participation in the survey after year one (*p* < 0.001). Further modeling with linear regressions of the year-one responses for all six items of the survey based on the participants' availability to follow-up in year two revealed no significant relationship. This finding that availability for a follow-up survey does not depend on year one responses provides qualitative evidence that the follow-up survey response data is missing at random (MAR), where the propensity for data to be missing is not inherent to the missing data, rather dependent on another variable (40). This supports the modeling approach that differences in missingness between the rural sites did not create different biases in the responses over time.

### Generalized Estimating Equations

Generalized Estimating Equations (GEE) were used to analyze changes in the measures of access to pet care resources over the study period. GEE is a statistical method used to analyze longitudinal data while considering multiple relevant covariates, even when the mathematical relationship between independent and dependent variables contains biased coefficients and parameter estimations (41, 42). GEE can account for individual and environmental variations that occur within repeated observations and controls for unobservable differences between individuals by allowing researchers to estimate the variation within individuals based on a few observations per individual (42, 43). This method of analysis is used for correlated data with binary, discrete, or continuous outcomes and is especially helpful when correlations are not specified/structured because it allows for selection of a correlation matrix when setting up the model (40, 43). Further benefits of using GEE include: the ability to appropriately handle time varying and time-invariant predictors; being more flexible with missing data compared to traditional repeated measure ANOVA's; and a robustness to the misspecification of the correlations structure (41, 43–45). In this study, the exchangeable correlation structure was employed so correlations between subsequent measures were assumed to be the same, regardless of the length of time of the interval (40). The main effects feature of GEE was utilized in this study to capture the nuanced relationship between one independent variable (e.g., preferred language, household income, study site) and the measures of access to pet care services at a specified time (46).

GEE analyses assessing how the presence or absence of PFL in the two urban and rural communities influences measures of access to pet care were conducted using SPSS version 25. The following independent factors were included in the model: preferred language, gender, age, ethnicity, household income, highest level of education completed, born in the U.S., study site, and survey date. The variables for preferred language were Spanish and “other,” with English being the reference category. Gender was measured as Female and “other,” with Male being the reference category. Age was measured in a range of years, including 18–30, 30–60 and a reference of 60 or older. The dichotomous variables for ethnicity included Latino/a, Black, and “other,” with White as the reference category. Household income was measured as $60,000 or more, $45,000–$60,000, $30,000–$45,000, $15,000–$30,000, with $0–15,000 as the reference category. Highest level of education was measured as college degree and high school degree or equivalent, with less than a high school degree as the reference category. Response options for the discrete variable, born in the U.S., were yes or no, with no serving as the reference category. For all demographic questions, “prefer not to answer” was provided as a response option. The variables for study site were the PFL intervention site (Madison, WI or Granger, WA) and comparison site (Seattle, WA or Wilder, ID), which provided the reference. Survey date was measured as a continuous variable. The demographic variables were included in the model because they could potentially affect the access to care outcome. Survey date is included to help analyze changes over time. GEE was then run for the dataset on the aggregated and disaggregated measure of access to pet resources. The disaggregated measures of access to pet care included six individual questions about affordability of services, geographic proximity to services, services in an individual's preferred language, and availability of pet healthcare information. Aggregate measures of access to pet resources were generated by taking the average of participants responses to all six of the questions. The negative numbers reported in the tables on GEE findings indicate lower access to care, while the positive numbers indicate higher access to care.

### Wilcoxon-Signed Rank Test

To integrate multiple lines of correlation, the influence of engagement with the PFL program on perceptions of access to pet care resources was also assessed for the subset of participants who were PFL clients in one of the two intervention sites (Madison, WI and Granger, WA). Data on the community members who engaged with the PFL program were transferred from PFL's client database into REDCap. Study participants were identified as PFL clients when there was a match between the address provided by the study participant and the address on file for the client in the PFL client database. Pre-intervention data were collected at the first time point, while post-intervention data were collected during the second year of data collection. The Wilcoxon-signed rank test was used to measure any changes in the six OHCA survey questions measuring perceptions of access to pet care between pre-intervention and post-intervention. The Wilcoxon-signed rank test was selected because it is a non-parametric statistical approach for within-group comparison. It is a paired-difference test, meaning repeated measurements on a single sample are compared to assess whether their population mean ranks differ (47).

## Results

### Propensity Score Matching

**Figure 1:**
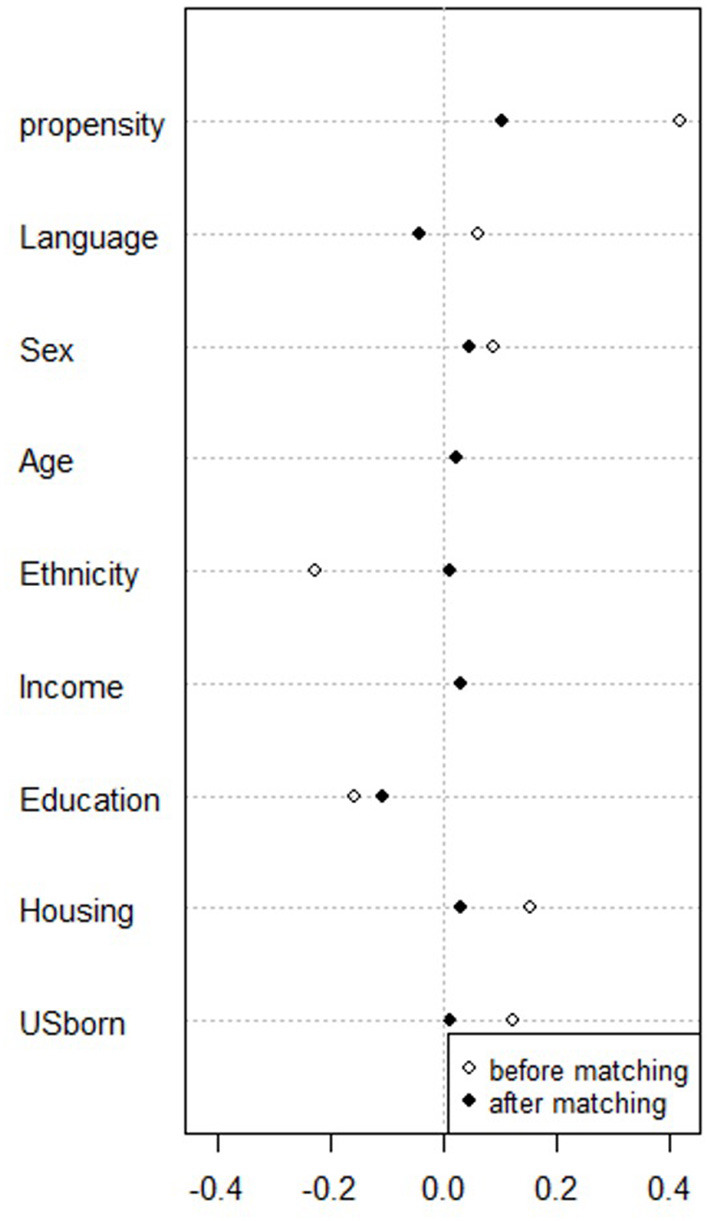
Dotplot of standardized mean differences (Cohen's *d)* for all covariates before and after matching survey participants in Madison, WI and Seattle, WA (*N* = 512).

**Figure 2:**
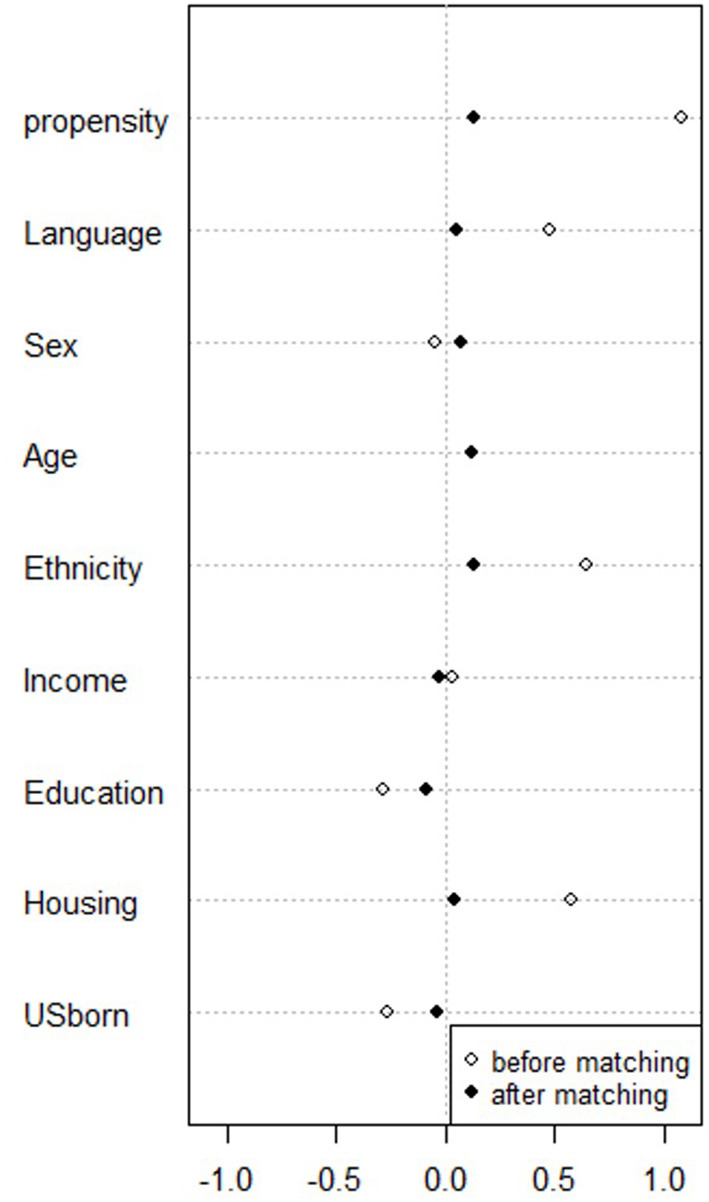
Dotplot of standardized mean differences (Cohen's *d)* for all covariates before and after matching survey participants in Granger, WA and Wilder, ID (*N* = 234).

Propensity score matching resulted in a final sample size of 512 participants from the urban sites and 234 participants from the rural sites (Table 2). Results of the overall balance test (48) are reported in Table 3. For both matched groups, no covariates demonstrated a large imbalance (|d| > 0.25). Figures 1, 2 present the standardized mean differences (Cohen's *d*) for all covariates before and after propensity score matching.

**Table 3:** Propensity score matching results of the overall balance test (48) for the intervention and comparison groups.

	**Chi-square**	**df**	***p*-value**
Urban sites	3.420	8.000	0.905
Rural sites	2.148	8.000	0.976

### Impacts of PFL on Overall Perceptions of Access to Pet Care

Results of the GEE analysis for the aggregate measure of access to pet care are presented in Table 4. The urban site that received the PFL intervention was associated with a higher aggregate measure of access to pet care compared to the urban site that does not have PFL present (*p* = 0.001). In the urban sites, people who spoke Spanish were associated with lower access to pet care than English speakers (*p* = 0.003). Participants who identified as Latino/a (*p* = 0.023) or an ethnicity categorized as “other” (*p* = 0.014) in the urban sites were associated with lower access to pet care than those who identified as White. The presence of PFL in a rural community did not have a statistically significant association with the aggregate measure of access to pet care. In the rural sites, people who were born in the U.S. were associated with higher access to pet care than individuals who were not born in the U.S. (*p* = 0.034). People with household incomes over $60,000 (*p* = 0.021), between $30,000 and $45,000 (*p* = 0.032), and between $15,000 and $30,000 (*p* = 0.043) were associated with higher access to pet care than people with a household income range of $0-$15,000.

**Table 4:** Generalized Estimating Equations to examine how the presence of PFL in an urban and rural community influences aggregated measures of perceived access to pet care.

	**Urban communities** **(*N* = 512)**	**Rural communities** **(*N* = 234)**
**Preferred language**		
Spanish	**−0.562 (−0.937**, **−0.186)**	−0.242
Other	−0.370	0
**Sex**		
Female	−0.012	0.118
Other	0.506	0
**Age**		
18–30 years old	0.024	−0.225
30–60 years old	0.053	−0.217
**Ethnicity**		
Other (Asian, Native American, multi-ethnic)	**−0.177 (−0.318**, **−0.035)**	0.001
Black	−0.038	0.054
Latino/a	**−0.256 (−0.475**, **−0.036)**	0.068
**Household income**		
> $60,000	0.095	**0.350 (0.052, 0.648)**
$45,000–$60,000	−0.094	0.161
$30,000–$45,000	−0.085	**0.319 (0.027, 0.610)**
$15,000–$30,000	0.032	**0.308 (0.010, 0.607)**
**Education**		
College degree	−0.089	0.136
High school degree or equivalent	−0.053	0.148
**Born in the U.S**.		
Yes	−0.155	**0.258 (0.020, 0.495)**
**Study site**		
PFL Intervention Site	**0.133 (0.052, 0.214)**	−0.079
Survey date	−0.073	0.112

*All significant findings are bolded (p < 0.05), and 95% confidence intervals are reported in parenthesis for all significant findings. The reference categories are described in greater detail in section Generalized Estimating Equations*.

### Impacts of PFL on Perceptions of the Individual Components of Access to Pet Care

Results of the GEE analysis for the disaggregated measures of access to pet care in the urban communities are presented in Table 5. The urban community with the PFL intervention was associated with higher access to affordable pet care options than the urban community without PFL present (*p* < 0.001). The urban community with PFL was associated with higher access to pet care service providers who offer payment options than the urban community without PFL (*p* < 0.001). The presence of PFL in an urban community was associated with lower access to pet care services in a participants' preferred language than the urban community without PFL present (*p* = 0.013). There were several demographic factors that also impacted the disaggregated measures of access to pet care services in the urban communities. Household incomes of $45,000-$60,000 were associated with lower access to affordable pet care options than household incomes of $0-$15,000 (*p* = 0.042). Those who identified their sex as “other” were associated with higher access to affordable pet care options than those who identified as male (*p* = 0.025). Participants with a college degree were associated with lower access to pet care service providers who offer payment options than participants with less than a high school degree (*p* = 0.044). Participants reported higher access to pet care services nearby earlier in the study period in comparison to later in the study period (*p* = 0.045). People who identified with an “other” ethnicity were associated with lower access to places nearby to buy pet supplies than people who identified as White (*p* = 0.016). Participants had higher access to places nearby to buy pet supplies earlier in the study period in comparison to later in the study (*p* = 0.001). Spanish speakers (*p* < 0.001) and those who spoke an “other” language (*p* < 0.001) were associated with lower access to pet care services in their preferred language than English speakers. Those who identified their sex as “other” were associated with higher access to pet care services in their preferred language than those who identified as male (*p* < 0.001). Participants who were 30–60 years old were associated with higher access to pet care services in their preferred language than participants who were more than 60 years old (*p* = 0.005). Those who identified as Latino/a (*p* = 0.006), Black (*p* = 0.027), or an “other” ethnicity (*p* = 0.015) were associated with lower access to pet care services in their preferred language than individuals who identified as White. Household incomes of >$60,000 (*p* = 0.023) or between $15,000 and $30,000 (*p* = 0.036) were associated with higher access to pet care services in their preferred language than household incomes of $0–$15,000. People born in the U.S. were associated with lower access to pet care services in their preferred language than people who were not born in the U.S. (*p* = 0.03). Spanish speakers (*p* = 0.04) and those who spoke an “other” language (*p* < 0.001) were associated with lower access to information for their pet's healthcare than English speakers. Those who identified their sex as “other” were associated with higher access to information for their pet's healthcare than those who identified as male (*p* = 0.025).

**Table 5:** Generalized Estimating Equations to examine how the presence of PFL in an urban community influences disaggregated measures of perceived access to pet care (*N* = 512).

	**Affordable options**	**Affordable options (payment plans)**	**Geographic proximity (pet care services)**	**Geographic proximity (pet supplies)**	**Preferred language**	**Pet healthcare information**
**Preferred language**						
Spanish	−0.495	−0.311	−0.478	−0.076	**−1.147** **(−1.723**, **−0.572)**	**−0.356** **(−0.695**, **−0.016)**
Other	0.155	0.125	0.056	−0.081	**−1.528** **(−2.283**, **−0.774)**	**−0.511** **(−0.797**, **−0.226)**
**Sex**						
Female	−0.049	0.000	−0.057	−0.047	0.028	0.020
Other	**0.971** **(0.120, 1.822)**	−0.378	0.174	0.116	**1.094** **(0.677, 1.511)**	**0.582** **(0.074, 1.090)**
**Age**						
18–30 years old	0.016	0.131	0.065	0.083	0.004	−0.008
30–60 years old	0.044	0.022	0.063	0.026	**0.155 (0.046, 0.265)**	0.059
**Ethnicity**						
Other (Asian, Native American, multi-ethnic)	−0.228	−0.226	−0.155	**−0.323** **(−0.586**, **−0.059)**	**−0.216** **(−0.391**, **−0.041)**	−0.013
Black	0.039	−0.017	0.057	−0.032	**−0.135** **(−0.255**, **−0.015)**	−0.104
Latino/a	−0.219	−0.277	−0.175	−0.205	**−0.408** **(−0.697**, **−0.120)**	−0.150
**Household income**						
> $60,000	−0.075	0.131	0.090	0.138	**0.177** **(0.024, 0.330)**	0.092
$45,000–$60,000	**−0.325** **(−0.637**, **−0.012)**	0.038	−0.115	−0.059	−0.059	0.012
$30,000–$45,000	−0.278	−0.213	−0.061	0.112	0.047	0.058
$15,000–$30,000	−0.154	0.115	−0.099	0.133	**0.187** **(0.012, 0.361)**	0.082
**Education**						
College degree	−0.208	**−0.298** **(−0.587**, **−0.009)**	−0.117	0.146	0.005	0.084
High school degree or equivalent	−0.054	−0.071	−0.061	0.145	−0.103	0.040
**Born in the U.S**.						
Yes	−0.194	0.036	−0.172	−0.144	**−0.234** **(−0.446**, **−0.023)**	−0.136
**Study site**						
Madison, WI	**0.342** **(0.191, 0.494)**	**0.340** **(0.169, 0.510)**	0.101	0.027	**−0.121** **(−0.217**, **−0.026)**	−0.003
Survey date	0.026	−0.101	**−0.140** **(−0.276**, **−0.003)**	**−0.174** **(−0.280**, **−0.068)**	**−0.109** **(−0.208**, **−0.010)**	−0.060

*Refer to Table 4 caption*.

Results of the GEE analysis for the disaggregated measures of access to pet care in the rural communities are presented in Table 6. The presence of the PFL intervention in a rural community did not have a statically significant association with any of the six disaggregated measures of access to pet care. However, there were several demographic factors that impacted the disaggregated measures of access to care in the rural communities. Participants who identified as Latino/a were associated with higher access to affordable pet care options than participants who identified as White (*p* = 0.046). Household incomes of >$60,000 were associated with higher access to affordable pet care options than individuals with a household income of $0-$15,000 (*p* = 0.015). Responses that occurred later in the study period were associated with higher access to pet care service providers who offered payment options in comparison to responses earlier in the study period (*p* = 0.002). Participants with a high school degree or equivalent were associated with higher access to pet care services nearby than participants with less than a high school education (*p* = 0.029), and Spanish speakers were associated with lower access to nearby places to buy pet supplies than English speakers (*p* = 0.011). Spanish speakers were associated with lower access to pet care services in their preferred language than English speakers (*p* = 0.037). People who were born in the U.S. reported higher access to pet care services in their preferred language than individuals who were not born in the U.S. (*p* = 0.014). Participants who identified as Latino/a were associated with higher access to information about pet's healthcare than individuals who identified as White (*p* = 0.038). A household income of $0-$15,000 was associated with lower access to information about pet's healthcare than household incomes >$60,000 (*p* = 0.002), between $45,000 and $60,000 (*p* = 0.011), and between $30,000 and $45,000 (*p* = 0.007). People who were born in the U.S. were associated with higher access to information about their pet's healthcare than individuals who were not born in the U.S. (*p* = 0.013).

**Table 6:** Generalized Estimating Equations to examine how the presence of PFL in a rural community influences disaggregated measures of perceived access to pet care (*N* = 234).

	**Affordable options**	**Affordable options (payment plans)**	**Geographic proximity (pet care services)**	**Geographic proximity (pet supplies)**	**Preferred language**	**Pet healthcare information**
**Preferred language**						
Spanish	−0.308	−0.283	−0.049	**−0.440** **(−0.778**, **−0.101)**	**−0.368** **(−0.713**, **−0.023)**	−0.156
**Sex**						
Female	0.167	−0.139	0.464	−0.052	0.038	0.024
**Age**						
18–30 years old	−0.039	0.207	−1.068	−0.101	−0.054	−0.158
30–60 years old	−0.050	−0.155	−0.740	−0.010	−0.056	−0.102
**Ethnicity**						
Other (Asian, Native American, multi-ethnic)	0.353	0.043	−0.303	0.075	−0.117	0.133
Black	0.084	0.297	−0.241	0.083	0.225	−0.070
Latino/a	**0.287** **(0.005, 0.568)**	0.185	−0.144	0.041	−0.096	**0.159** **(0.009, 0.310)**
**Household income**						
> $60,000	**0.499** **(0.099, 0.899)**	0.153	0.409	0.021	0.244	**0.365** **(0.131, 0.599)**
$45,000–$60,000	0.119	0.099	0.140	0.112	0.046	**0.406** **(0.094, 0.718)**
$30,000–$45,000	0.326	0.415	0.349	0.033	0.153	**0.304** **(0.083, 0.526)**
$15,000–$30,000	0.357	0.411	0.567	0.219	0.092	0.178
**Education**						
College degree	0.064	−0.193	0.307	0.205	0.260	0.226
High school degree or equivalent	0.036	0.038	**0.522** **(0.052, 0.991)**	−0.021	0.101	0.083
**Born in the U.S**.						
Yes	0.230	0.155	0.378	−0.006	**0.335** **(0.068, 0.601)**	**0.179** **(0.037, 0.321)**
**Study site**						
Granger, WA	−0.148	0.003	−0.169	0.119	−0.006	−0.024
Survey date	0.033	**0.284** **(0.107, 0.460)**	0.121	0.080	0.070	0.050

*Refer to Table 4 caption*.

### Impacts of PFL Client Status on Perceptions of the Individual Components of Access to Pet Care

Results of the Wilcoxon signed-rank test demonstrated that participants in Madison, WI (*N* = 37) had higher perceptions of access to affordable pet cares services after becoming a PFL client in comparison to before they were a PFL client (*p* = 0.027). In Granger, WA (*N* = 61) the results of this test revealed that participants had higher perceptions of access to pet care services in their preferred language after becoming a PFL client in comparison to before they were a PFL client (*p* = 0.048) (Table 7).

**Table 7:** Wilcoxon-signed rank test to examine perceptions of access to pet care pre-intervention and post-intervention for PFL clients.

	***p*-value**	**Negative ranks**	**Positive ranks**
**Madison (*****N*** **=** **37)**			
Affordable options	**0.027**	0	6
Affordable options (payment plans)	0.221	1	4
Geographic proximity (pet care services)	0.157	0	2
Geographic proximity (pet supplies)	0.739	3	3
Preferred language	0.783	3	2
Pet Healthcare information	0.102	0	3
**Granger (*****N*** **=** **61)**			
Affordable options	0.296	3	7
Affordable options (payment plans)	0.118	2	6
Geographic proximity (pet care services)	0.571	4	6
Geographic proximity (pet supplies)	0.586	3	6
Preferred language	**0.048**	1	7
Pet healthcare information	0.165	1	6

*p-values are bolded to indicate significant findings (p < 0.05)*.

## Discussion

The findings of this study demonstrate how a program that focuses on addressing the structural barriers to accessing pet support services (e.g., affordability, geographic proximity, availability of services in an individual's preferred language) can drive community-wide changes in perceptions of the accessibility of services. This study builds on previous research that found when structural barriers to accessing pet care services were addressed through a community-level intervention, the individual-level factor of pet owners' race and ethnicity were not a primary determinant for seeking pet support services (13). The development and validation of the OHCA, which includes a subset of questions to assess perceptions of access to pet support services, represents a potentially significant advancement in the animal welfare field's ability to develop and evaluate programs that can address historic and ongoing exclusion of marginalized populations. To our knowledge, this study is the first to measure the impacts of a pet support program on community members' perceptions of four dimensions of access to care. A detailed discussion of the measured impacts of PFL on the four dimensions of access to care identified during the development of the OHCA are included below.

### Affordability

Cost of services is the most frequently cited barrier to accessing pet support services [e.g., (7–9, 11–19)]. In this study, PFL was associated with higher access to affordable pet care services at both the community-level and in the pre-and post-intervention analyses for the urban site (Madison, WI). PFL's programming focuses on addressing affordability of pet support services by offering no or low-cost procedures (e.g., spay and neuter), services (e.g., microchips), medications (e.g., vaccines and de-worming treatment), and supplies (e.g., food, treats, litterboxes, collars, and leashes) in historically underserved communities, providing training and mentorship support to animal service organizations, and engaging in policy advocacy on the national level to increase the understanding of how systemic poverty impacts pet owners. Unfortunately, some of the literature has undermined efforts to address affordability of services by associating a pet owner's willingness to pay for services with the strength of their emotional attachment to their companion animal (49–51). This narrative has reinforced implicit bias against individuals living in poverty and justifies the assertion that pet ownership is—or should be—reserved for individuals who can afford all aspects of pet ownership under all circumstances (17, 20, 24, 52). In contrast, PFL engages in their work with historically underserved communities through a social justice perspective that asserts that pet ownership should be available to anyone who wishes to access the benefits of the human-animal bond (53). This program philosophy aligns with more recent research that has discussed other problematic systemic factors contributing to high costs of veterinary care, such as an increase in veterinary education program costs (54), an increased demand for veterinary healthcare services that mimic those offered in the human healthcare field (20), the disproportionate growth between cost and pet owners' perceived worth of services (20), and economic downturns (24). Within this framework that recognizes the broader community-level factors driving the lack of affordability of services, some animal welfare programs are advocating for, and modeling, a shift in the definitions of “minimum acceptable level of caretaking” and “upmost level of medicine and surgery” in the veterinary medicine profession (24). Future research could gain greater insights into the findings of the present study by examining which specific components of the PFL program drive the greatest improvements in perceptions of the affordability of care.

Perceived availability of payment options to pay for care were also higher in the urban intervention site (Madison, WI) in comparison to the site without PFL. The option to utilize different payment options is often cited as a deciding factor for which service provider a pet owner chooses (55). While possession of pet health insurance is one approach that has increased pet owner spending for veterinary care, it has not been documented as having a significant impact on the frequency of veterinary visits (56). Other programs being piloted to address the affordability of pet support services by offering alternative payment options include “Pet Health Care Credit Cards” (20), “pay what you can” models (20, 57), or subsidizing basic preventive care (e.g., spay/neuter, vaccinations). While there are some concerns that these alternate payment systems could negatively impact the revenue of private veterinarians, initial research indicates that many of the clients who utilize these alternate payment options were not previously utilizing any veterinary care services (54).

### Geographic Proximity

In this study, there were no significant differences in perceptions of proximity to pet care services or pet supplies stores between the intervention and comparison communities. The negative impacts of transportation barriers on service utilization have been well-documented in historically marginalized communities (58). Previous research indicates that geographic proximity to pet support service providers is an important factor in a pet owner's ability to obtain care for their pet (7–9, 13, 18, 55, 59). To explain this issue, Cromley and McLafferty (60) discuss the concept of “distance decay,” in which as an individual's cost, time, and effort increase, their willingness and ability to travel to access care decreases. The intention of the PFL program is not to create new service providers in the community, but instead to connect community members with services that already exist outside of the focus area. PFL does this by proving transportation for pets and their owners to and from appointments and offering to deliver no-cost pet supplies (e.g., food, treats, litterboxes, collars, and leashes) directly to people's homes. Another strategy to overcome this barrier are mobile clinic models, but they are largely offered infrequently and unpredictably (21). Rauktis et al. (25) proposed the alternative strategy of hosting both pet and human food bank events in a common location to promote greater access to basic pet supplies for vulnerable populations. Future research could assess how these approaches or other strategies help overcome the barrier of geographic proximity to care.

### Preferred Language

In this study, the urban community with the PFL intervention was associated with lower access to pet care services in the pet owners' preferred language than the comparison community. However, this particular finding may have been driven by a relative lack of language diversity present in the intervention site (Madison, WI), while residents of the comparison site (Seattle, WA) were documented as speaking a much wider range of languages, including Spanish, Cambodian, Vietnamese, Russian, Chinese, Japanese, and Albanian. In contrast, this study found an increase in perceptions of access to pet care services in the pet owner's preferred language for the pre-intervention and post-intervention analyses in the rural intervention site (Granger, WA). This was an important programmatic finding, given a significant portion of the sample in the rural sites reported their preferred language as Spanish (Table 2). While there is some research indicating that availability of services in the pet owner's preferred language is a barrier to accessing veterinary care (7, 9), this body of evidence is significantly less robust than other components of accessibility that were explored in this study. PFL works to address language-related barriers by employing bilingual staff members and providing written materials (e.g., fliers, information sheets) in multiple languages. These findings may indicate a need for additional research to identify strategies that would have a greater measurable impact for overcoming language-based barriers to care. Future research should also explore how both cultural and linguistic considerations in discussing animal ethics and care practices inform the perceived accessibility of services (7, 61).

### Pet Healthcare Information

In this study, there were no measurable changes in perceived access to information for the intervention sites. PFL potentially addresses this dimension of access by serving as a non-veterinary source of information that strives to be both knowledgeable and trustworthy. Their service providers focus on providing thorough explanations of a pet care procedure/visit and ensuring they address any concerns of the pet owner before providing transport to the appointment. These findings may indicate a need for additional research to identify strategies that would have a greater measurable impact for overcoming language-based barriers to care. Identified sources of information for pet owners include veterinarians, veterinary technicians, animal shelter professionals, animal control officers, non-veterinary animal experts, friends/family members, the internet, and advertisements/campaigns (8, 19, 62–65). Concerns about the credibility of pet care information that is obtained from online sources and non-veterinarian professionals has led to an increased value placed on information obtained from a veterinarian (63, 64). However, several studies have discussed challenges associated with obtaining information from veterinarians, including a lack of cultural competence training in the veterinary profession, feeling as though the veterinarian does not have time to answer additional questions, concern that by asking for additional information the veterinarian will think the client did not listen close enough to the information previously given, or fear that disclosing that they use online sources to find pet health information will harm the client's relationship with the veterinarian (16, 61, 64, 65). Additionally, some pet owners express a distrust of veterinary professionals, including believing that veterinarians are promoting preventative products and services for financial gain and believing that their veterinarian lacks education on alternate pet healthcare options (9, 17, 62). This lack of trust of veterinarians as a source of information may result in different levels of understanding the importance for routine veterinary care that result in less desirable trajectories of pet health (7). Future research should examine how accessing information regarding pet care through sources other than veterinarians impacts pet health outcomes and how client misperceptions of veterinarians' advice can be improved.

### Limitations and Future Directions

Several limitations should be considered when interpreting the findings of this study. First, it is important to note that the correlations observed in this study between the presence of PFL in a community and higher perceived access to care are not evidence of direct causation. It is possible that the observed differences between the intervention and comparison communities could be driven by community-level differences that existed before PFL was present and/or developed during the study period, such as differences in baseline pet ownership rates (35) or demographic differences (e.g., cultural, linguistic) between the sites that were not controlled for within the original site matching criteria. The site matching criteria limited the study sites to communities with high rates of poverty and high racial/ethnic diversity, which limits the generalizability of these findings to communities with differing demographic profiles. Propensity score matching was employed to control for the demographic differences between individuals in the intervention and comparison communities in this study. However, with the reduced sample size that resulted from propensity score matching, there is potential the urban and rural samples may not be representative of the demographic profile of the entire study sites' zip code. Replicating this study in additional communities served by PFL could improve the animal welfare field's understanding of the extent to which these findings are generalizable to other communities.

Additionally, while multiplicity of testing can potentially result in type 1 errors, the exploratory nature of this study encouraged multiple tests to measure the impacts of “having access” to services and “gaining access” to services (2, 66). The primary focus was on exploring how the presence of PFL in a community (but not necessarily direct engagement with PFL as a client) impacts perceptions of access to pet care. While data were collected on individuals in the community who specifically engaged with PFL as clients, a small number of clients in the available sample size for the study period limited statistical power for conducting the GEE analysis using this sample. To address potential false positives, exploratory analyses were conducted using the Wilcoxon signed-rank test and presented in this study to provide an initial assessment of the influence of PFL client status on the measures of access to care (Table 7). Although not all statistically significant, almost every one of the disaggregate measures of access to care increased after a study participant became a PFL client. This is a promising indicator that the observed differences in the present study might also be detectable at the individual level when statistical power is sufficient. Of note, more statistically significant findings were generated in the datasets with more statistical power. The number of separate analyses performed within the current study, however, creates the possibility of type 1 error, therefore the relationships identified in this study should be further examined in future research. Future studies should expand upon analyses of how engagement with a pet supportive intervention or awareness of the program affect perceptions of access to pet care and attempt to isolate which of the specific components of the PFL model create the highest impacts on perceived access to care and pet health and welfare outcomes. Furthermore, given the structural nature of the issue of access to pet support care, driving significant changes in perceptions of access likely requires more than just 2 years of programming. Future studies might consider longitudinally measuring the impacts of programs designed to address access to care issues over a longer period of time to assess if any changes in perceived access to care occur and are sustained.

### Conclusion

Together, these findings provide some of the first evidence that effective pet support programming aiming to increase the accessibility of services for historically marginalized populations must engage communities with recognition of the variety of both individual and structural barriers they might experience.

## Data Availability Statement

The raw data supporting the conclusions of this article will be made available by the authors, without undue reservation.

## Ethics Statement

The studies involving human participants were reviewed and approved by University of Denver IRB protocol 1234950. Written informed consent for participation was not required for this study in accordance with the national legislation and the institutional requirements.

## Author Contributions

TH, SH, KE, JW, and KM: conceptualization and original draft preparation. KE and JW: literature review. SH, TH, AA, SN, and KM: additions, edits, and review. All authors have read and agreed to the published version of the manuscript.

## Funding

This study was funded by Maddie's Fund (Grant # 37613), the Arnall Family Foundation (formerly WaterShed Animal Fund) (Grant # 37685), and the William and Charlotte Parks Foundation (Grant #38023). TH's Research Fellowship is funded through a grant provided by an anonymous donor to the University of Denver's Graduate School of Social Work. SH and KMs' positions are partially funded by the latter's American Humane Endowed Chair research fund.

## Conflict of Interest

The authors declare that the research was conducted in the absence of any commercial or financial relationships that could be construed as a potential conflict of interest.

## Publisher's Note

All claims expressed in this article are solely those of the authors and do not necessarily represent those of their affiliated organizations, or those of the publisher, the editors and the reviewers. Any product that may be evaluated in this article, or claim that may be made by its manufacturer, is not guaranteed or endorsed by the publisher.

## Abbreviations

CBRA: Community-Based Research Assistant
GEE: Generalized Estimating Equations
OHCA: One Health Community Assessment
MCAR: Missing Completely at Random
MAR: Missing at Random
PFL: Pets for Life
PSM: Propensity Score Matching
SPSS: Statistical Package for the Social.
